# Severe symptomatic acute hyponatremia in traumatic brain injury responded very rapidly to a single 15 mg dose of oral tolvaptan; a Mayo Clinic Health System hospital experience – need for caution with tolvaptan in younger patients with preserved renal function

**DOI:** 10.15171/jrip.2017.05

**Published:** 2016-11-01

**Authors:** Macaulay Amechi Chukwukadibia Onuigbo, Nneoma Agbasi

**Affiliations:** ^1^Department of Nephrology, Mayo Clinic Health System, Eau Claire, WI 54702, USA; ^2^North East London NHS Foundation Trust, UK

**Keywords:** Hyponatremia, Renal function, Subarachnoid hemorrhage, Syndrome of inappropriate, anti- diuretic hormone (SIADH), Tolvaptan

## Abstract

Tolvaptan is now well established as a potent pharmaceutical agent for symptomatic hyponatremia from syndrome of inappropriate antidiuretic hormone secretion (SIADH), congestive heart failure and liver cirrhosis. Previous studies had recruited older (63-65 years) patients with mild renal impairment (serum creatinine, 1.3-1.4 mg/dl). A 2012 report in the *Journal of Neurology, Neurosurgery & Psychiatry* described tolvaptan as a "lifesaving drug". A major outcome concern in the treatment of chronic hyponatremia is potentially fatal pontine demyelination from over-rapid correction of serum sodium >0.5 mEq/dL/h. The maximum reported correction of serum sodium within 24 hours was 13 mEq/L in a case of SIADH. We recently experienced the dramatic correction of hyponatremia at 1 mEq/dL/h over 18 hours, following 15 mg of oral tolvaptan in a 32-year old male patient with normal kidney function (serum creatinine 0.76 mg/dL), following traumatic brain injury (TBI). Tolvaptan is indeed an effective and life-saving drug for post-TBI hyponatremia. However, we strongly recommend the use of lower doses of tolvaptan (≤15 mg/d) in younger patients with more preserved renal function to avoid the development of life-threatening pontine demyelination.

**Implication for health policy/practice/research/medical education:**

We report a 32-year-old man who presented with progressively symptomatic hyponatremia due to the SIADH secretion complicating TBI. He responded very briskly to a low dose of the arginine vasopressin antagonist tolvaptan with normalization of hyponatremia within 18 hours from 121 mmol/L to 139 mmol/ L. His young age and normal kidney function (serum creatinine of 0.76 mg/dL, eGFR of 126 mL/min/1.73 sq. m BSA) contrasts sharply with the historical clinical trials during drug development of Tolvaptan which studied older patients with often stage III chronic kidney disease (CKD). This case report implicated that the dose of oral tolvaptan in younger patients with preserved renal function would be lower than the currently recommended standard doses of the drug. Furthermore tolvaptan should be dosed singly, with repeat daily doses only prescribed and administered after physician evaluation of response of hyponatremia to initial dose.

## Introduction

Hyponatremia, the most commonly encountered electrolyte abnormality, affects as many as 30% of hospitalized patients (1,2). It is a powerful predictor of poor outcomes, especially in patients with congestive heart failure or cirrhosis (3,4). In traumatic brain injury (TBI), hyponatremia results from either the syndrome of inappropriate anti diuretic hormone (SIADH) secretion or from cerebral salt wasting. The failure to excrete electrolyte-free water from the persistent secretion of ADH despite low serum osmolality usually underlies the development of hyponatremia in SIADH. The resultant decrease in plasma osmolality following hyponatremia causes intracellular edema, a potentially fatal condition for the brain where cell swelling exceeding 8% will pose a serious risk for brainstem herniation (5).

The arginine vasopressin antagonist, tolvaptan, is now well established as a potent pharmaceutical agent to treat symptomatic hyponatremia from SIADH (6-9). Tolvaptan is an orally active antagonist of arginine vasopressin type 2 receptors in the collecting duct of the kidney that inhibits water reabsorption without substantially affecting the electrolyte balance. According to the analysis of the SALT 1 and SALT 2 trials, in patients with euvolemic or hypervolemic hyponatremia, tolvaptan was effective in increasing serum sodium concentrations at day 4 and day 30 at doses of 30 mg daily or higher (6-8). Nevertheless, there remains the concern over too rapid correction of (chronic) hyponatremia, since potentially life-threatening pontine demyelination may result if the rate of correction exceeded a rate >0.5 mEq/dL/h. Our review of the tolvaptan-hyponatremia literature revealed the maximum reported correction of serum sodium within 24 hours was 13 mEq/dL (126 to 139) in a 51-year old female with one year history of SIADH and 12 mEq/dL (124 to 136) in a 47-year old Caucasian man who had developed severe symptomatic hyponatremia (124 mEq/dL) following severe head injury from an assault that was complicated by Enterobacter cloacae meningoencephalitis (10,11). In both cases, the correction followed 15 mg of oral tolvaptan (10,11). We recently experienced the dramatic correction of serum sodium by 18 mEq/L with serum sodium increasing from 121 mEq/dL to 139 mEq/dL over 18 hours, following the administration of a lone single dose of 15 mg oral tolvaptan in a 32-year old Caucasian male patient with normal kidney function. Hyponatremia was secondary to SIADH following TBI.

## Case Presentation

**Figure 1:**
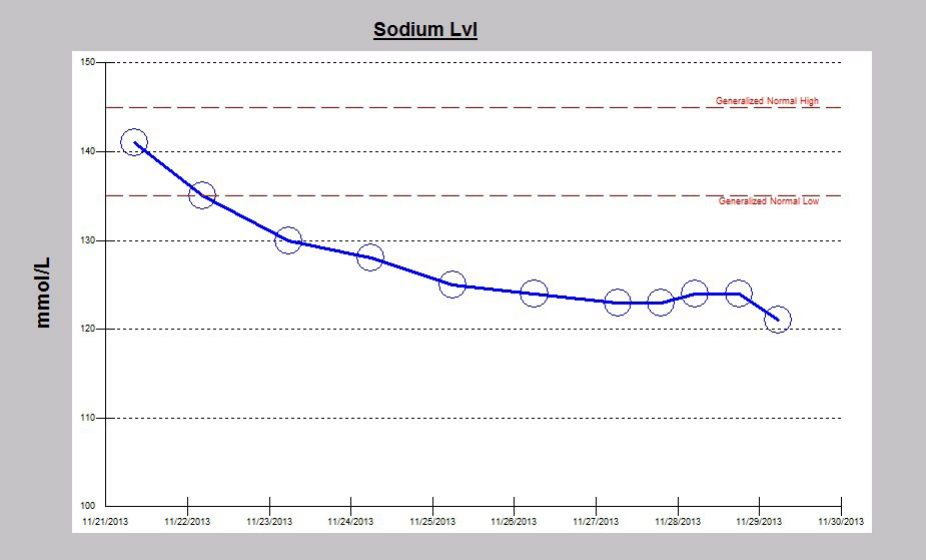
Falling serum Na despite fluid restriction, oral Na tablets and repeated infusions of 3% saline boluses

**Figure 2:**
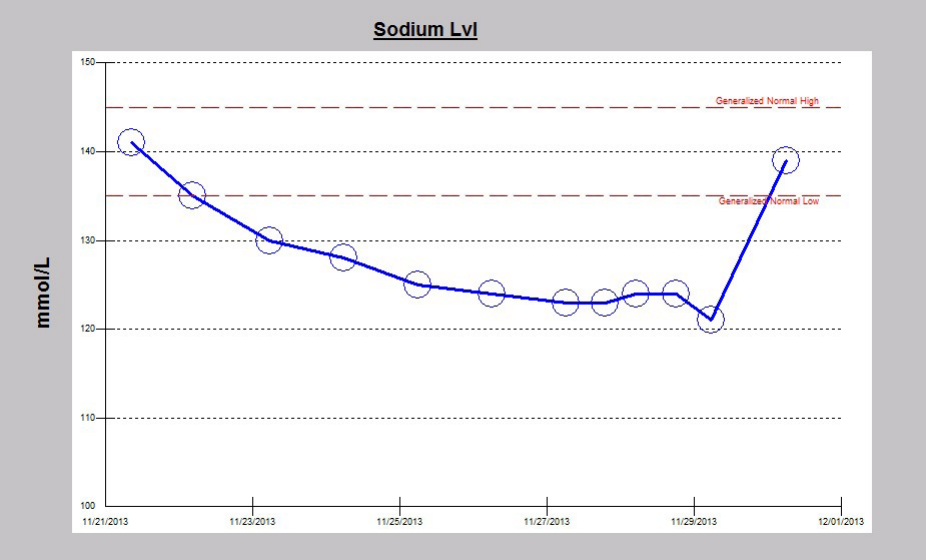
Rapidly normalized serum Na despite fluid liberalization and a single oral dose of 15 mg tolvaptan

**Figure 3:**
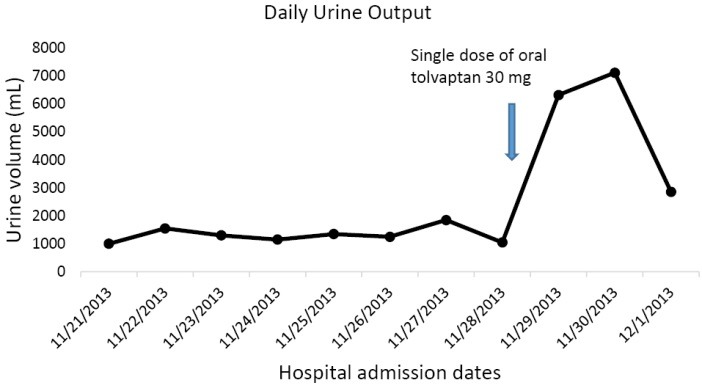
Polyuria following single dose of 15 mg oral tolvaptan.

**Figure 4:**
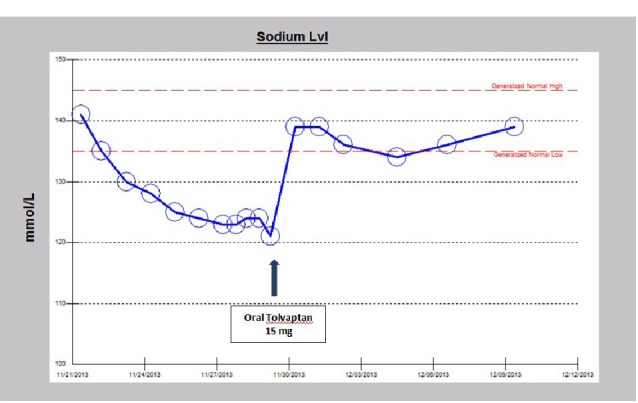
Normalized serum Na 11 days following a single oral dose of 15 mg tolvaptan

A 32-year-old Caucasian male patient was recently admitted to the trauma service of our hospital in following a garbage truck motor vehicle accident with a semi-trailer truck, with the patient on the passenger side of the truck. The patient had altered level of consciousness at the scene and was transported to Mayo Clinic Health System Eau Claire Emergency Room as a level I trauma activation. Significant injuries included acute right posterior 11th and 12th rib fractures, large scalp hematoma, and a small focus of acute intracranial hemorrhage near the vertex of the skull on CT-scan, thought to represent subarachnoid hemorrhage. The patient was intubated earlier on for airway protection. Serum sodium on admission was normal at 141 mEq/dL. Serum sodium fell during the hospital stay and despite infusions of intermittent timed 250 cc boluses of 3% saline, fluid restriction to 800 cc/day and the use of oral sodium chloride tablets, the serum sodium continued to fall (Figure 1). The patient was seen as a nephrology consult for rapidly worsening acute symptomatic hyponatremia. Symptoms of acute hyponatremia included blurred vision, poor memory and cognitive impairment. Since the plan was to start oral tolvaptan, fluid restriction was discontinued, fluid intake was liberalized and the patient was educated on the potential effects of tolvaptan therapy. The patient received a single dose of 15 mg oral tolvaptan, given at 1229 pm on the consult day, and was not to receive the next day’s dose until after nephrology review. Soon after the tolvaptan tablet administration, urine output quickly increased, the patient noticed increased thirst and was soon drinking “tons of water”. Serum sodium had rapidly increased to 139 mEq/dL, an increase of 18 mEq in 18 hours, a very rapid rate of correction of 1 mEq/dL/h (Figure 2). Simultaneously, the daily urine output had more than quintupled from 1050 cc to 6325 cc the next day (Figure 3). Tolvaptan was promptly discontinued. Concurrent arginine vasopressin level was 1.4 pg/mL, still inappropriately high for a concomitant serum sodium of 121 mEq/dL, consistent with SIADH. Two days after the single oral tolvaptan dose, the symptoms of blurred vision, poor memory and cognitive impairment had fully resolved and he was discharged home. A post-hospitalization evaluation, 11 days post-injury, revealed an otherwise normal patient, but with still persistent left gaze diplopia. Serum sodium was stable at 139 mEq/dL (Figure 4).

## Discussion

Most of the studies on tolvaptan for the treatment of symptomatic (chronic) hyponatremia as in the SALT-1, SALT-2, EVEREST and SALTWATER trials had recruited older patients, age range 64-67 years, with some degree of renal impairment, baseline serum creatinine of about 1.3-1.4 mg/dL (6-8,12). Doses of tolvaptan utilized in these studies were 30 mg daily dose or higher. The very rapid correction that we observed in our young patient following just a single oral dose of 15 mg of tolvaptan and the subsequent sustained effect of this single dose after eleven days of the single dose administration is extraordinarily remarkable and is the first such report in the literature. No such rapid correction has been previously reported. The maximum reported correction of serum sodium within 24 hours in the literature, as previously recounted were 12 mEq/dL and 13 mEq/dL, respectively, following 15 mg dose of oral tolvaptan (10,11). Of note, these were relatively younger patients, aged 47 years and 51years, respectively; however the renal functional status of both case reports were not reported (10,11). Indeed, the 51-year old female with one year history of SIADH developed symptomatic hypotension from >6.5 L overnight polyuria following the 15 mg dose of oral tolvaptan that ultimately she required as little as 3 mg daily dose of tolvaptan on discharge (11). Compared to most other patients studied previously, our patient was much younger, aged 32 years, and had normal renal function (serum creatinine, 0.76 mg/dL), with an estimated glomerular filtration rate (eGFR) of 126 mL/min/1.73 sq. m BSA. We suggest the use of lower doses of tolvaptan (15 mg or lower) in younger patients with preserved kidney function, more so when treating for chronic hyponatremia, to mitigate against the development of potentially fatal pontine demyelination.

## Acknowledgments

This work is dedicated to the memory of a very dear friend, Ikechukwu Ojoko (Idejuogwugwu), who passed away back home in Port Harcourt, Nigeria, some years ago, after a reported brief illness. Idejuogwugwu, you are truly missed. This work is also dedicated to the memories of the 153 Nigerians who died in a fiery plane crash in Lagos, Nigeria, on June 3, 2012. May their souls rest in perfect peace. Finally, we dedicate this work to the memory of late Professor Dimitrios Oreopoulos; he was indeed a great teacher and mentor to the first author.

## Authors’ contribution

MACO; conception, design, acquisition of data, data analysis, interpretation of data, literature review, drafting the article and final approval of manuscript. NA; literature review, drafting the article and final approval of manuscript.

## Conflicts of interest

The authors report no conflicts of interest. The authors alone are responsible for the content and writing of the article.

## Ethical considerations

Ethical issues (including plagiarism, data fabrication, double publication) have been completely observed by the authors. Written consent was obtained from the patient for publication of the study.

## Funding/Support

None.
